# The clinically approved drugs dasatinib and bosutinib induce anti-inflammatory macrophages by inhibiting the salt-inducible kinases

**DOI:** 10.1042/BJ20141165

**Published:** 2015-01-06

**Authors:** James Ozanne, Alan R. Prescott, Kristopher Clark

**Affiliations:** *MRC Protein Phosphorylation and Ubiquitylation Unit, College of Life Sciences, Sir James Black Centre, University of Dundee, Dundee DD1 5EH, Scotland, U.K.; †Cell Signaling and Immunology, College of Life Sciences, Sir James Black Centre, University of Dundee, Dundee DD1 5EH, Scotland, U.K.

**Keywords:** bosutinib, chemical biology, dasatinib, inflammation, interleukin-10, salt-inducible kinase, Btk, Bruton’s tyrosine kinase, CREB, cAMP-response-element-binding protein, CRTC, CREB-regulated transcription co-activator, DMEM, Dulbecco’s modified Eagle’s medium, ERK 1/2, extracellular-signal-regulated kinase 1/2, GAPDH, glyceraldehyde-3-phosphate dehydrogenase, HA, haemagglutinin, HDAC, histone deacetylase, IKK, IκB kinase, IκB, inhibitor of NF-κB, JNK, c-Jun N-terminal kinase, IL, interleukin, IRF, interferon regulatory factor, LPS, lipopolysaccharide, MAPK, mitogen-activated protein kinase, M-CSF, macrophage colony-stimulating factor, MSK, mitogen- and stress-activated protein kinases, NF-κB, nuclear factor κB, Pam_3_CSK_4_, tripalmitoylcysteinylseryl-(lysyl)_4_, PGE_2_, prostaglandin E_2_, qPCR, quantitative real-time PCR, SIK, salt-inducible kinases, TBK1, TANK-binding kinase 1, TLR, Toll-like receptor

## Abstract

Macrophages switch to an anti-inflammatory, ‘regulatory’-like phenotype characterized by the production of high levels of interleukin (IL)-10 and low levels of pro-inflammatory cytokines to promote the resolution of inflammation. A potential therapeutic strategy for the treatment of chronic inflammatory diseases would be to administer drugs that could induce the formation of ‘regulatory’-like macrophages at sites of inflammation. In the present study, we demonstrate that the clinically approved cancer drugs bosutinib and dasatinib induce several hallmark features of ‘regulatory’-like macrophages. Treatment of macrophages with bosutinib or dasatinib elevates the production of IL-10 while suppressing the production of IL-6, IL-12p40 and tumour necrosis factor α (TNFα) in response to Toll-like receptor (TLR) stimulation. Moreover, macrophages treated with bosutinib or dasatinib express higher levels of markers of ‘regulatory’-like macrophages including LIGHT, SPHK1 and arginase 1. Bosutinib and dasatinib were originally developed as inhibitors of the protein tyrosine kinases Bcr-Abl and Src but we show that, surprisingly, the effects of bosutinib and dasatinib on macrophage polarization are the result of the inhibition of the salt-inducible kinases. Consistent with the present finding, bosutinib and dasatinib induce the dephosphorylation of CREB-regulated transcription co-activator 3 (CRTC3) and its nuclear translocation where it induces a cAMP-response-element-binding protein (CREB)-dependent gene transcription programme including that of IL-10. Importantly, these effects of bosutinib and dasatinib on IL-10 gene expression are lost in macrophages expressing a drug-resistant mutant of salt-inducible kinase 2 (SIK2). In conclusion, our study identifies the salt-inducible kinases as major targets of bosutinib and dasatinib that mediate the effects of these drugs on the innate immune system and provides novel mechanistic insights into the anti-inflammatory properties of these drugs.

## Abstract 2

We have discovered that bosutinib and dasatinib, which are protein tyrosine kinase inhibitors used in the clinic to treat human cancer, induce anti-inflammatory but block pro-inflammatory cytokine production by inhibiting the serine/threonine kinases known as the salt-inducible kinases.

## INTRODUCTION

Drug development for the treatment of chronic inflammatory diseases has mainly focused on interfering with the pro-inflammatory pathways driving the acute inflammatory response. There is now an increasing appreciation that the resolution of inflammation is a carefully co-ordinated process whose failure contributes to the pathogenesis of human diseases such as rheumatoid arthritis, systemic lupus erythematosus and psoriasis [1]. Understanding the molecular networks controlling the terminal phase of inflammation could reveal new strategies for the treatment of inflammatory and autoimmune diseases.

An important aspect of the resolution of inflammation is the reprogramming of macrophage fate from a pro-inflammatory classically activated phenotype to an anti-inflammatory phenotype [2–4]. The ability of macrophages to rapidly adapt to their microenvironment has led to the description of a myriad of different macrophage populations with overlapping but distinct properties. One group of anti-inflammatory macrophages is the ‘regulatory’-like macrophages induced by cAMP-elevating agonists such as prostaglandin E_2_ (PGE_2_), immune complexes, glucocorticoids and interleukin (IL)-10 [4]. This macrophage population may have potent immunomodulatory functions as it produces large quantities of the anti-inflammatory cytokine IL-10 while producing very low levels of pro-inflammatory mediators such as tumour necrosis factor α (TNFα) and IL-12 in response to activation of Toll-like receptors (TLRs) [4]. This macrophage population also expresses a number of specific markers including SPHK1 and LIGHT [5], which distinguishes them from ‘alternatively activated’ macrophages induced by IL-4 which express FIZZ and YM1 [6]. Notably, the administration of ‘regulatory’-like macrophages to mice alleviates the cardinal features of different inflammatory diseases including sepsis and experimental autoimmune encephalomyelitis [7,8]. A potential therapeutic strategy for the treatment of chronic inflammatory and autoimmune diseases could therefore involve the administration of compounds inducing the formation of ‘regulatory’-like macrophages to promote the resolution of inflammation.

TLR stimulation provides the first signal for the activation of macrophages, which induces the phosphorylation and activation of the mitogen-activated protein kinases (MAPKs), nuclear factor κB (NF-κB) and interferon regulatory factor (IRF) signalling axes [9,10]. The overall phenotype of the macrophage is then directed by secondary signals that regulate cAMP-response-element-binding protein (CREB) function [10]. This transcription factor drives the production of many anti-inflammatory molecules. It follows that agonists that suppress CREB function, such as IFNγ (interferon γ) [11], induce classically activated macrophages, whereas activation of CREB, by agents such as PGE_2_ [12], promotes the formation of ‘regulatory’-like macrophages. A central problem in this area is therefore to identify strategies to activate CREB-dependent gene transcription in macrophages.

Recently, we made a major breakthrough by identifying the first series of structurally unrelated small-molecule inhibitors that induce several characteristic features of ‘regulatory’-like macrophages [13]. Mechanistic studies revealed that these compounds inhibit members of the salt-inducible kinases (SIKs) family to promote CREB function in macrophages [13]. Inhibition of the SIKs leads to the dephosphorylation of CREB-regulated transcription co-activator 3 (CRTC3), and its translocation to the nucleus where it associates with CREB to drive a gene expression programme including that of IL-10. Importantly, the effects of the pan-SIK inhibitor HG-9-91-01 were lost in cells expressing a drug-resistant mutant of SIK2 [13]. Inhibition of the SIKs also leads to the suppression of pro-inflammatory cytokines IL-6, IL-12 and TNFα. The combined action of SIK inhibitors on anti-inflammatory and pro-inflammatory cytokines in macrophages suggests that these compounds may have important advantages over current therapies used for the treatment of inflammatory diseases.

To gain greater insight into the therapeutic potential of targeting the SIKs for the treatment of chronic inflammatory diseases, we aimed to identify new potent inhibitors of these kinases that are currently used in the clinic. In contrast with all the other members of the AMP-activated protein kinase (AMPK) family, which possess a large hydrophobic residue at the gatekeeper site, the SIKs have a small threonine residue at this position within its catalytic domain. The SIKs share this feature with many members of the protein tyrosine kinases. Since several clinically approved drugs for the treatment of cancer target various protein tyrosine kinases including Bcr-Abl and Src [14], we hypothesized that one or more of these compounds might also inhibit the SIKs *in vivo*, thereby opening the possibility of repurposing these therapeutics for the treatment of chronic inflammatory diseases. In the present study, we report the exciting discovery that both bosutinib and dasatinib potently inhibit the SIKs leading to the formation of ‘regulatory’-like macrophages. Our study provides new insights into the molecular mechanisms underpinning the anti-inflammatory properties of dasatinib and proposes a new strategy for the treatment of chronic inflammatory diseases.

## MATERIALS AND METHODS

### Materials

HG-9-91-01 was kindly provided by Nathanael Gray (Harvard, Boston, MA, U.S.A.). Bosutinib, dasatinib, erlotinib, gefitinib, lapatinib and vandetinib were purchased from Selleck Chem, whereas imatinib and sunitinib were obtained from LC Labs. Protein kinase inhibitors were dissolved in DMSO and stored at −20°C as 10 mM solutions. Tripalmitoylcysteinylseryl-(lysyl)_4_ (Pam_3_CSK_4_), was from Invivogen and lipopolysaccharide (LPS) (*Escherichia coli* O55:B5) was from Alexis Biochemicals. Mouse recombinant macrophage colony-stimulating factor (M-CSF) was purchased from R&D Systems.

### Antibodies

An antibody against a non-phosphorylated peptide of human CRTC3 (CWKEEKHPGFR, S277D) used for immunoprecipitation and antibodies against the pSer^370^ peptide (RLFSLpSNPSLST, S253D) and pSer^162^ peptide (LNRTNpSDSALH, S369D) of human CRTC3 used for immunoblotting were provided by the Division of Signal Transduction Therapy, University of Dundee and have been previously described [13]. The following commercially available antibodies were used for immunoblotting:- horseradish peroxidase-conjugated secondary antibodies (Pierce), anti-α-tubulin (Sigma), anti-haemagglutinin (HA) (Roche), anti-IκB kinase (IKKβ, where IβB is inhibitor of NF-βB) (Millipore), anti-glyceraldehye-3-phosphate dehydrogenase (GAPDH), anti-pSer^133^ CREB, anti-pThr^581^ mitogen- and stress-activated protein kinases (MSK1), anti-pSer^177/181^ IKKβ, anti-TANK-binding kinase 1 (TBK1), anti-pSer^172^ TBK1, anti-pSer^933^ p105, anti-pSer^177/181^ IKKβ, anti-pSer^396^ IRF3, anti-p38α/β MAPK, anti-pThr-Gly-pTyr sequences of extracellular-signal-regulated kinase 1/2 (ERK1/2) and p38 MAPKs, anti-IκBα, anti-pTyr^207^ CRKL, anti-pTyr^416^ Src and anti-pTyr^223^ Bruton's tyrosine kinase (BTK) (Cell Signaling Technology) and anti-pThr-Pro-pTyr sequence of c-Jun N-terminal kinase (JNK) 1/2 (Invitrogen). For immunofluorescence staining, the anti-CRTC3 was obtained from Abcam, whereas Alexa Fluor® 594 conjugated anti-rabbit IgG was obtained from Invitrogen.

### Cell culture

Primary macrophages were generated by differentiating bone marrow from 6- to 12-week-old C57BL/6 mice for 7 days in Dulbecco's modified Eagle's medium (DMEM) supplemented with 5 ng/ml recombinant M-CSF (R&D systems), 2 mM glutamine, 10% FBS, penicillin and streptomycin. Bone-marrow-derived macrophages were differentiated on non-tissue-culture-treated plastic, harvested and replated at a density of 100000 cells/cm^2^ per 0.1 ml on tissue culture-treated plastic in fresh medium before stimulation on day 8. RAW264.7 cells were cultured in DMEM containing 10% FBS, 2 mM glutamine and penicillin and streptomycin. RAW264.7 cell lines expressing wild-type and the drug-resistant mutant of SIK2 under a tetracycline responsive promoter were as previously described [13]. Cells were treated for 1 h with or without inhibitors and then stimulated for up to 24 h with either 1 μg/ml Pam_3_CSK_4_ or 100 ng/ml LPS.

### Gene expression analysis

mRNA was extracted from cells using the MicroElute Total RNA kit following the manufacturers’ instructions (Omega Bio-Tek). cDNA was generated from 0.5 μg of total RNA using the iScript cDNA synthesis kit and quantified by quantitative real-time PCR (qPCR) using the SsoFast EvaGreen Supermix on a CFX384 real-time system (Bio-Rad Laboratories). The relative expression of each gene was calculated from *C*_T_ values using the Pfaffl method [15] and was normalized against the mRNA levels of housekeeping genes including 18S RNA and GAPDH. Results are reported relative to untreated control cells, which were set to 1.

### Cytokine secretion

The concentration of TNFα, IL-6, IL-10 and IL-12p40 in cell-free culture supernatants were measured using the Bio-Plex Pro Assay system (Bio-Rad).

### Immunoblotting

Cells were extracted in lysis buffer [50 mM Tris/HCl, pH 7.4, 1 mM EDTA, 1 mM EGTA, 50 mM NaF, 5 mM sodium pyrophosphate, 10 mM sodium β-glycerol 1-phosphate, 1 mM DTT, 1 mM sodium orthovanadate, 0.27 M sucrose, 1% (v/v) Triton X-100, 1 μg/ml aprotinin, 1 μg/ml leupeptin and 1 mM PMSF]. Cell extracts were clarified by centrifugation and protein concentrations were determined using the Bradford assay. To immunoprecipitate endogenous CRTC3, 1 mg of cell extract was incubated with 5 μg of anti-CRTC3 [13] covalently cross-linked on to Protein G–Sepharose. Immune complexes were washed three times with lysis buffer, eluted in 1% SDS and separated by SDS/PAGE. To detect proteins in cell lysates, 20 μg of protein extract was separated by SDS/PAGE. After transfer to PVDF membranes, proteins were detected by immunoblotting and visualized by treating the blots with ECL (GE Healthcare) followed by autoradiography.

### Immunofluorescence

Macrophages were seeded on glass coverslips and treated with inhibitors for 1 h. Cells were then fixed for 10 min in 4% paraformaldehyde, permeabilized for 5 min using 0.1% Triton X-100 in PBS and blocked for 45 min using 3% BSA in PBS. Cells were stained with anti-CRTC3 and anti-tubulin followed by Alexa Fluor® 596-conjugated anti-rabbit and Alexa Fluor® 488-conjugated anti-mouse antibodies respectively. Antibodies were diluted in blocking buffer and incubated with the cells for 45 min. Cells were mounted in Prolong Gold Antifade Reagent containing DAPI (Invitrogen) and visualized under a Zeiss LSM 700 confocal microscope using an Alpha Plan-Apochromat ×100/numerical aperture (NA) 1.46 objective (optical section thickness 0.7 μm). Fields of cells were selected at random using only the DAPI stained channel. Optical sections were taken through the centre of the nuclei, 10 fields collected per coverslip and images were quantified using the Volocity programme (PerkinElmer). Nuclei were identified using the DAPI stained channel, merged objects were separated and the mean intensity of the green channel (Alexa Fluor® 488) in the nuclear and cytoplasmic regions was measured. For each field, the mean of the nuclear to cytoplasmic intensity ratio was calculated, which was subsequently used to determine the overall mean nuclear to cytoplasmic intensity ratio for each treatment.

### Kinase assays

Recombinant wild-type and drug-resistant mutants of SIK1, SIK2 and SIK3 were expressed as GST-fusion proteins in HEK293 cells and purified on a glutathione–Sepharose column. BTK (amino acids 2–659) and Src (amino acids 1–536) were expressed in Sf9 insect cells with an N-terminal His6-tag and affinity purified on a Ni^2+^-nitrilotriacetate–agarose column. GST–Abl (amino acids 118–535) was co-expressed in bacteria with His–YopH and purified on glutathione–Sepharose. For the IC_50_ curve measurements, kinase assays were performed as described by Hastie et al. [16]. The following peptides were used as substrates: SIKs, ALNRTSSDSALHRRR; Abl, EAIYAAPFAKKK; Btk and Src, KVEKIGEGTYGVVYK. Kinase profiling was performed as previously described [17] and performed by the International Centre for Kinase Profiling (http://www.kinase-screen.mrc.ac.uk).

### Statistical analysis

Quantitative data are presented as the means±S.E.M. For experiments using primary macrophages, cultures were established from *n* mice. Each macrophage population was independently differentiated, cultured and stimulated before analysing the biological material. For experiments using the RAW264.7 macrophage cell line, *n* describes replicate culture wells in a single experiment. Statistical significance of differences between experimental groups was assessed using one-way or two-way ANOVA with the Bonferroni post-hoc test or a one-sample Student's *t* test when appropriate. Unless otherwise indicated, the data are compared with cells stimulated with TLR agonists in the absence of any drug. Differences in means were considered significant if *P*<0.05; *P<0.05, **P<0.01, ***P<0.001. ns refers to non-significant.

## RESULTS

### Identification of bosutinib and dasatinib as inhibitors of the SIKs

We tested a panel of clinically approved tyrosine kinase inhibitors used for treating various human cancers for their ability to also inhibit SIK2 and SIK3 *in vitro*. The selected compounds had a range of potencies against SIK2 and SIK3 with a set of compounds showing low nanomolar IC_50_ values whereas other compounds were completely inactive even at very high concentrations (100 μM) (Table 1). Notably, bosutinib and dasatinib potently inhibited all three SIK isoforms with IC_50_ values in the low nanomolar range. Both bosutinib and dasatinib inhibit a number of protein tyrosine kinases in addition to the SIKs (Supplementary Table S1). The potency of these drugs towards the SIKs is in a range similar to that for different protein tyrosine kinases targeted for the treatment of cancer including Bcr-Abl and Src (Table 2).

**Table 1:** IC_50_ (nM) for clinically approved cancer drugs against the different SIK isoforms

Drug	SIK1	SIK2	SIK3
Bosutinib	<3	<3	18
Dasatinib	<3	<3	10
Erlotinib		1600	4000
Gefitinib		800	3800
Imatinib		>100000	>100000
Lapatinib		>100000	>100000
Sorafenib		>100000	>100000
Sunitinib		400	11000
Vandetinib	370	120	740

**Table 2:** IC_50_ values (nM) for bosutinib and dasatinib against protein tyrosine kinases

Drug	Abl	BTK	Src
Bosutinib	1	15	2
Dasatinib	<1	1	2

### Bosutinib and dasatinib induce high IL-10 production in macrophages

**Figure 1:**
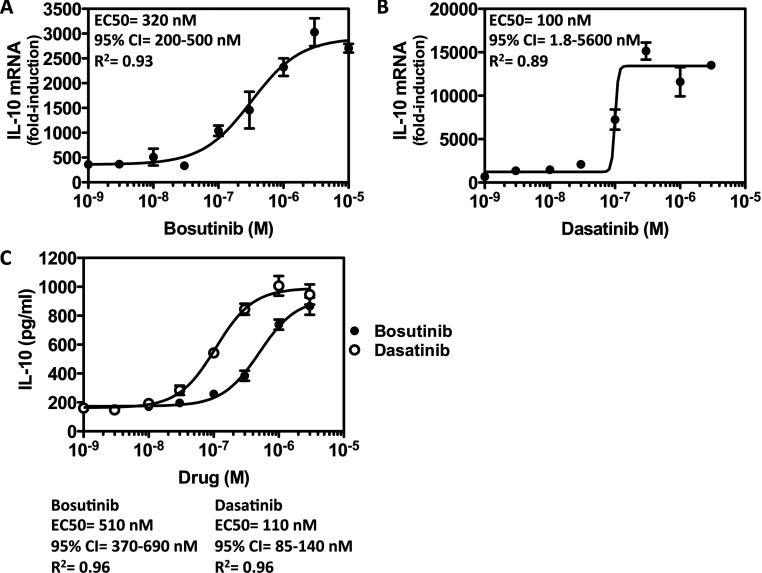
Bosutinib and dasatinib elevate IL-10 mRNA production and secretion in a dose-dependent manner Bone-marrow-derived macrophages were treated with the indicated concentrations of (**A**) bosutinib and (**B**) dasatinib for 1 h and then stimulated with LPS for 1 h. IL-10 mRNA levels were measured by qPCR (*n*=4, mean±S.E.M.). (**C**) Experiment was performed as above except culture supernatants were harvested after stimulation with LPS for 2 h. The concentration of IL-10 secreted into the culture medium was measured using the BIOPLEX system (*n*=4, mean±S.E.M.).

**Figure 2:**
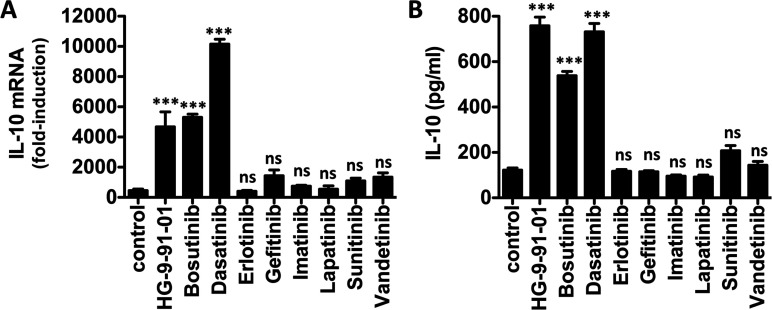
Bosutinib and dasatinib but not other protein tyrosine kinase inhibitors elevate IL-10 production in macrophages Bone-marrow-derived macrophages were treated with vehicle control or the indicated compounds for 1 h and then stimulated with LPS for (**A**) 1 h to induce mRNA expression or (**B**) 2 h for cytokine secretion. IL-10 mRNA levels were measured by qPCR, whereas the concentration of IL-10 secreted into the culture medium was measured using the BIOPLEX system (*n*=4, mean±S.E.M.). All compounds were used at 10 μM except HG-9-91-01, bosutinib and dasatinib which were used at 0.5, 3 and 0.3 μM respectively.

We reported previously that SIK inhibitors dramatically induce the production of IL-10 in macrophages stimulated with different TLR agonists [13], a key feature of ‘regulatory’-like macrophages. Consistent with the ability of bosutinib and dasatinib to inhibit the SIKs *in vitro*, these compounds induced the production of IL-10 in a dose-dependent manner (Figure 1). Pre-treatment of primary macrophages with bosutinib and dasatinib raised the mRNA levels (Figures 1A and 1B) and secretion (Figure 1C) of IL-10 in macrophages stimulated with the TLR4 agonist, LPS. However, dasatinib was around 10-fold more potent than bosutinib in elevating IL-10 production in macrophages. When used at optimal doses, dasatinib (300 nM) and bosutinib (3 μM) induced IL-10 production to similar levels as HG-9-91-01 (500 nM) in LPS-stimulated macrophages (Figure 2). In contrast, all other compounds (erlotinib, gefitinib, imatinib, lapatinib, sunitinib and vandetinib) which were shown to either not inhibit the SIKs, or only very weakly, failed to elevate IL-10 production even at very high doses (10 μM) (Figure 2).

### Bosutinib and dasatinib induce CRTC3 dephosphorylation and CREB-dependent gene transcription

**Figure 3:**
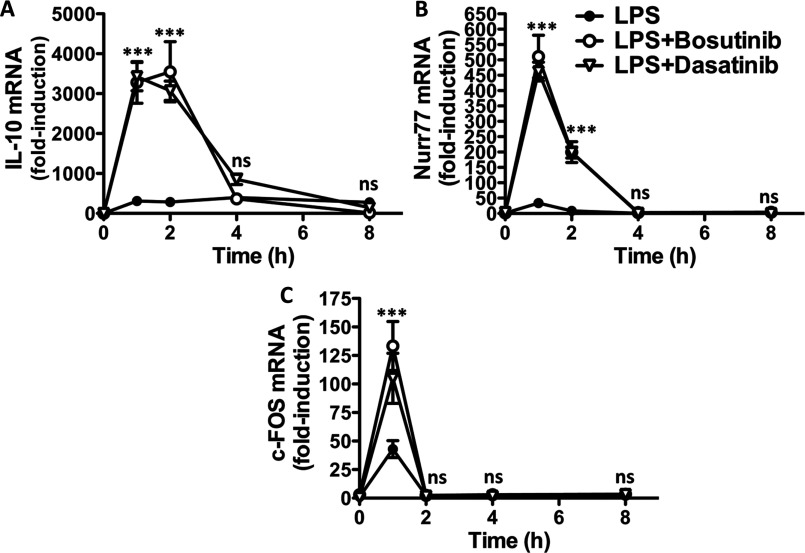
Bosutinib and dasatinib induce a gene expression programme directed by the transcription factor CREB Bone-marrow-derived macrophages were treated with vehicle control, 3 μM bosutinib or 0.3 μM dasatinib for 1 h and then stimulated with LPS for the indicated times. Expression of different CREB-dependent genes, (**A**) IL-10, (**B**) nurr77 and (**C**) c-FOS, were measured by qPCR. mRNA levels were normalized to 1 in unstimulated cells (mean±S.E.M., *n*=4).

We previously found that inhibition of the SIKs in primary macrophages not only induces IL-10 mRNA synthesis but also that of many other genes which all have in common a binding element for the transcription factor CREB within their promoter [13]. Consistent with the effects of dasatinib and bosutinib on macrophage polarization being the result of SIK inhibition, both compounds elevated the transcription of the CREB-dependent genes IL-10, Nurr77 and c-FOS in response to LPS or Pam_3_CSK_4_ (Figure 3 and Supplementary Figure S1). The time course of transcription of these immediate early genes was similar, characterized by a dramatic burst within the first hour of stimulation and coming back down near basal levels within 2–4 h.

**Figure 4:**
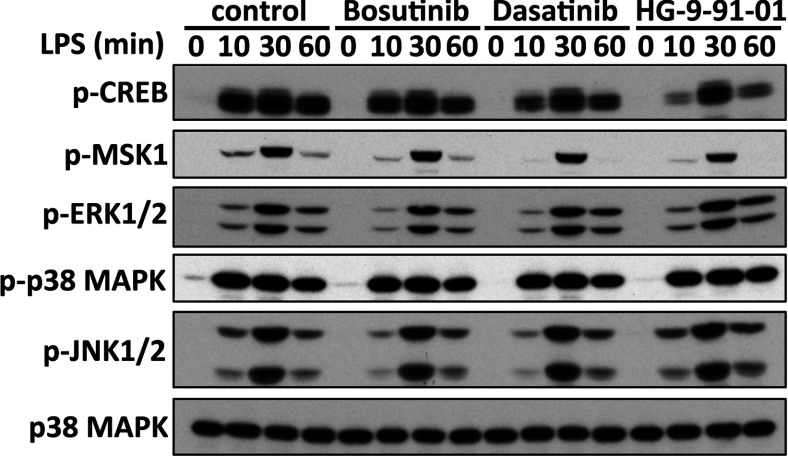
Bosutinib and dasatinib do not affect the TLR-stimulated phosphorylation of CREB Bone-marrow-derived macrophages were treated with vehicle control, 3 μM bosutinib, 0.3 μM dasatinib or 0.5 μM HG-9-91-01 for 1 h and then stimulated with LPS for 0, 10, 30, or 60 min. Cell lysates were immunoblotted using the indicated antibodies.

**Figure 5:**
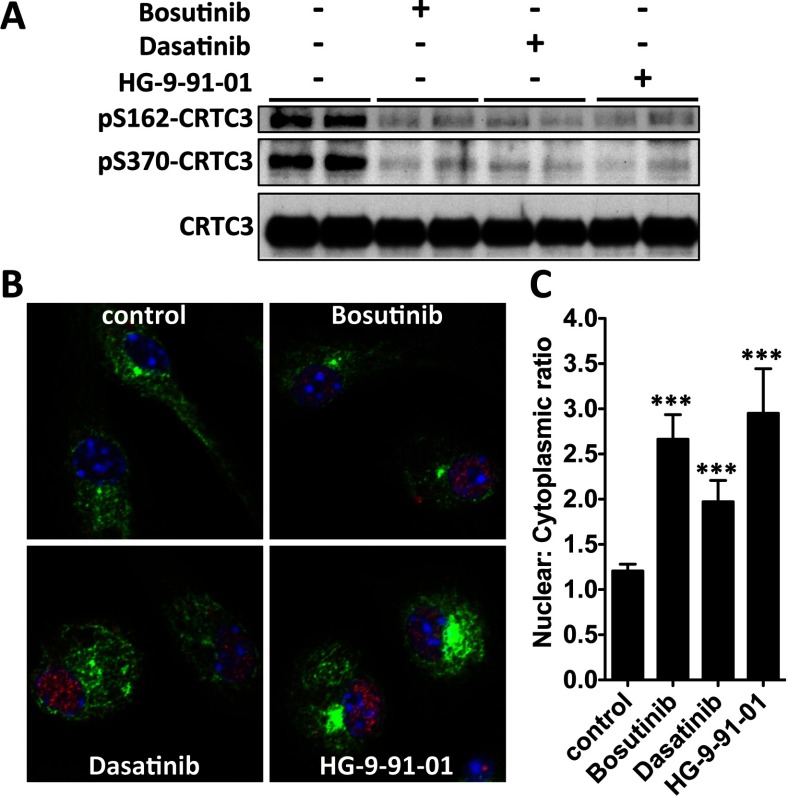
Bosutinib and dasatinib induce the dephosphorylation of CRTC3 and its nuclear translocation (**A**) RAW264.7 macrophages were treated with bosutinib, dasatinib, HG-9-91-01 or vehicle control for 1 h. CRTC3 was immunoprecipitated and phosphorylation of Ser^162^ and Ser^370^ was monitored by immunoblotting. (**B**) Bone-marrow-derived macrophages were plated on glass coverslips, and treated with bosutinib, dasatinib, HG-9-91-01 or vehicle control for 1 h. Samples were stained for CRTC3 (Alexa Fluor® 594), tubulin (Alexa Fluor® 488) and DNA (DAPI) content and imaged under a confocal microscope. (**C**) Quantification of CRTC3 nuclear translocation as a function of its nuclear to cytoplasmic ratio. (mean±S.E.M., *n*=10).

CREB activity is regulated by direct phosphorylation at Ser^133^ mediated by the kinases MSK1/2 [18] and via interactions with co-activators called CRTCs [19]. Bosutinib and dasatinib did not affect the pathways leading to the phosphorylation of CREB. Treatment of macrophages with bosutinib and dasatinib had no effect on the kinetics of activation of the different MAPKs including ERK1/2, and p38α/β in response to LPS or Pam_3_CSK_4_ (Figure 4 and Supplementary Figure S2). Moreover, these drugs did not affect the subsequent activation of MSK1/2 by ERK1/2 and p38α/β MAPKs or the ability of MSK1/2 to phosphorylate CREB at Ser^133^ in macrophages stimulated with TLR agonists. Instead, treatment of macrophages with bosutinib and dasatinib led to the dephosphorylation of the SIK substrate CRTC3 at Ser^162^ and Ser^370^ (Figure 5A) and its translocation to the nucleus (Figure 5B) where CRTC3 can promote CREB-dependent gene transcription.

### Expression of a drug-resistant mutant of SIK2 blocks the effects of bosutinib and dasatinib on IL-10 production

**Figure 6:**
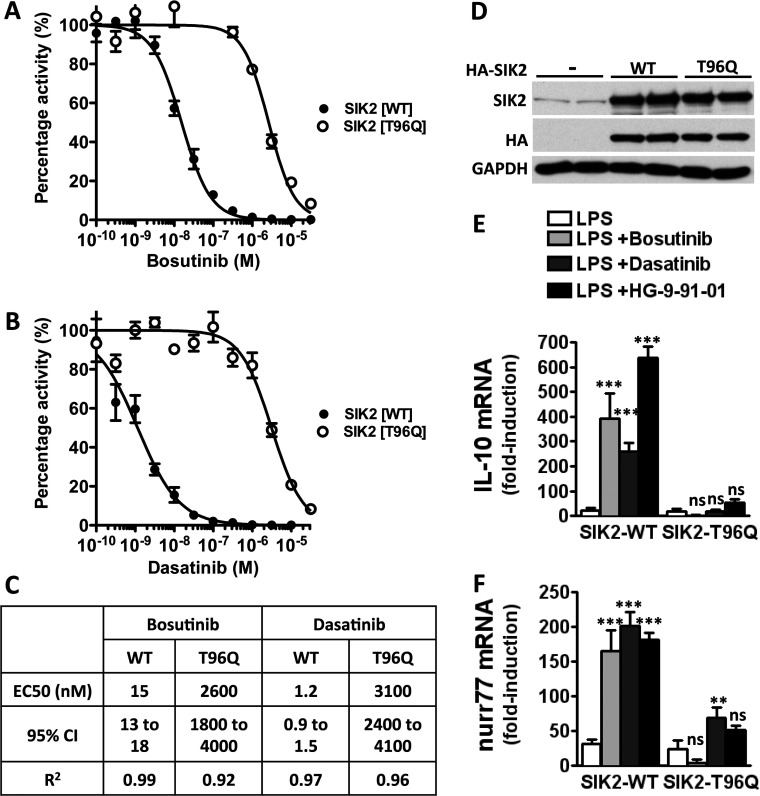
Bosutinib and dasatinib fail to induce IL-10 production in macrophages expressing a drug-resistant mutant of SIK2 The SIK2[T96Q] mutant is insensitive to (**A**) bosutinib and (**B**) dasatinib *in vitro*. Kinase assays were performed using purified SIK2 wild-type and SIK2[T96Q] in the presence of increasing amounts of inhibitors as described in the Materials and methods section (mean±S.D., *n*=2). (**C**) Statistical analysis of IC_50_ curves generated in (**A**) and (**B**). (**D**) RAW264.7 macrophages engineered to express HA-SIK2-WT and HA-SIK2[T96Q] were treated with doxycycline for 16 h, lysed and immunoblotted with the indicated antibodies. Cells transduced with a retrovirus containing no insert was used as negative control (−). (**E** and **F**) RAW264.7 macrophages expressing HA-SIK2-WT and HA-SIK2[T96Q] were treated with bosutinib, dasatinib, HG-9-91-01 or vehicle control for 1 h and then stimulated with LPS for 1 h. mRNA expression of IL-10 (**E**) and nurr77 (**F**) were measured by qPCR (mean±S.E.M., *n*=4). WT, wild-type.

The induction of bosutinib and dasatinib resistance in chronic myeloid leukaemia arises from the appearance of the T315I mutation in Bcr-Abl [14]. Mutation of the equivalent residue in SIK2, threonine 96, to a bulky amino acid such as leucine, phenylalanine, glutamic acid, lysine, methionine or glutamine rendered the enzyme resistant to KIN-112 and HG-9-91-01 [13]. Since the SIK2[T96Q] mutation was slightly more resistant than the other mutants to these drugs [13], we tested whether this mutant would also be resistant to bosutinib and dasatinib. Although bosutinib inhibited wild-type SIK2 *in vitro* with an IC_50_ value of 15 nM, bosutinib inhibited the SIK2[T96Q] mutant with an IC_50_ value of 2600 nM (Figures 6A and 6C). Similarly, dasatinib potently inhibited wild-type SIK2 with an IC_50_ value of 1.2 nM which increased to 3100 nM in the SIK2[T96Q] mutant (Figure 6C). These results clearly demonstrate that the inhibition of the SIKs by bosutinib and dasatinib are as a consequence of the presence of a small threonine residue at the gatekeeper site and mutation of this residue to a bulky glutamine renders the enzyme 100–1000-fold less sensitive to these drugs.

We subsequently engineered RAW264.7 macrophages to express wild-type and the drug-resistant mutant of SIK2 under the control of a promoter containing a tetracycline-responsive element [13]. Addition of doxycycline (a long-lived tetracycline analogue) to the culture medium induced high levels of protein expression of both wild-type and T96Q mutant of SIK2 and these levels were several-fold higher than the expression levels of endogenous SIK2 (Figure 6D). Bosutinib and dasatinib could strongly potentiate the LPS-stimulated CREB-dependent gene transcription including IL-10 and Nurr77 in cells expressing wild-type SIK2, but these drugs had little effect on the expression of these genes in macrophages expressing the drug-resistant SIK2[T96Q] mutant (Figures 6E and 6F). These results demonstrate that bosutinib and dasatinib influence IL-10 production by inhibiting the SIKs and not via the inhibition of the protein tyrosine kinases.

### Bosutinib and dasatinib promote key features of ‘regulatory’-like (IL-10^high^, IL-12^low^, TNFα^low^, SPHK1^high^, LIGHT^high^) macrophages

**Figure 7:**
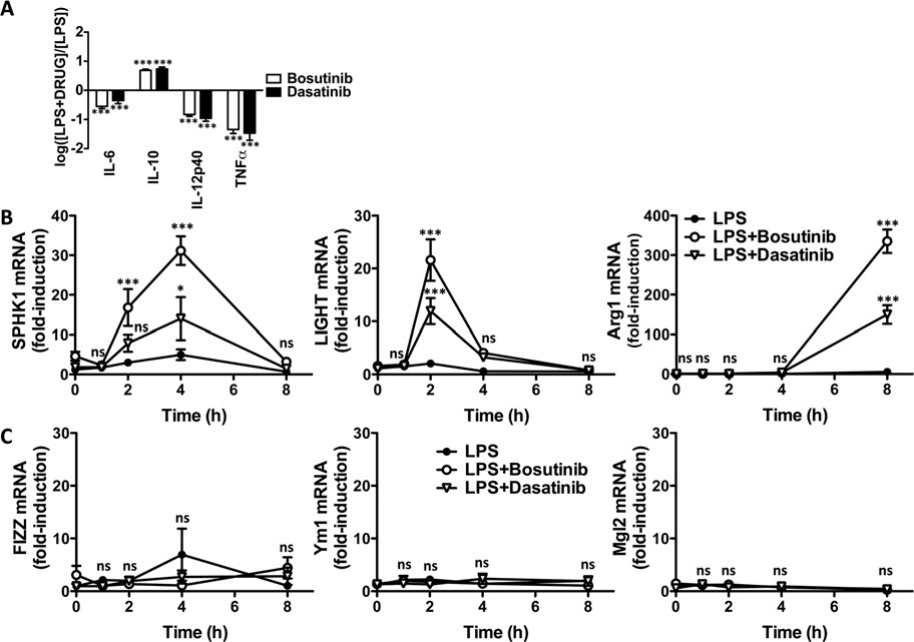
Bosutinib and dasatinib induce key features of ‘regulatory’ macrophages (**A**) Effect of bosutinib and dasatinib on cytokine secretion. Bone-marrow-derived macrophages were treated with vehicle control, 3 μM bosutinib or 0.3 μM dasatinib for 1 h and then stimulated with LPS for 8 h. The concentration of the different cytokines in the culture supernatant was measured using the BIOPLEX system. The data are depicted as the fold-change in cytokine secretion in the presence of the drug (*n*=4, mean±S.E.M.). Statistical significance was determined by comparing each dataset to 0 using a one-sample Student's *t* test. (**B**) Effect of bosutinib and dasatinib on the expression of markers of ‘regulatory’ macrophages. Bone-marrow-derived macrophages were treated with vehicle control, 3 μM bosutinib or 0.3 μM dasatinib for 1 h and then stimulated with LPS for the indicated times. Expression of LIGHT, SPHK1 and arginase 1 were measured by qPCR. mRNA levels were normalized to 1 in unstimulated cells (mean±S.E.M., *n*=4). (**C**) Effect of bosutinib and dasatinib on the expression of markers of alternatively activated macrophages. Experiment was performed as described in (**B**) but measuring the mRNA levels of FIZZ, Ym1 and Mgl2 (mean±S.E.M., *n*=4).

‘Regulatory’-like macrophages are also characterized by the production of low levels of pro-inflammatory cytokines and the expression of markers such as SPHK1, LIGHT and arginase 1 [4]. Interestingly, treatment of macrophages with bosutinib and dasatinib inhibited the production of IL-6, IL-12p40 and TNFα induced by LPS stimulation (Figure 7A; Supplementary Figure S3). Moreover, bosutinib and dasatinib elevated the expression of SPHK1, LIGHT and arginase 1 (Figure 7B) without affecting the expression of FIZZ, YM1 and Mgl2 (Figure 7C), which are markers of alternatively activated (M2a) macrophages generated in response to IL-4. Similar results were obtained when macrophages were stimulated with Pam_3_CSK_4_, a TLR1/2 agonist (Supplementary Figure S4). Our results demonstrate that bosutinib and dasatinib, like other SIK inhibitors such as MRT199665 and HG-9-91-01 [13], drive the polarization of macrophages towards a ‘regulatory’-like phenotype.

## DISCUSSION

In the present study, we show that the clinically approved drugs bosutinib and dasatinib control macrophage polarization by inhibiting the SIKs. Notably, treatment of macrophages with bosutinib or dasatinib, like other inhibitors of the SIKs [13], induced the formation of ‘regulatory’-like macrophages characterized by the production of high levels of IL-10 and very low levels of pro-inflammatory cytokines such as TNFα, IL-6 and IL-12. Moreover, bosutinib and dasatinib elevated the expression of *bona fide* markers of ‘regulatory’ macrophages including SPHK1, LIGHT and arginase 1 without affecting the expression of markers of alternatively activated macrophages such as FIZZ, Ym1 and Mgl2. Bosutinib and dasatinib were originally developed as drugs to block the activity of the protein tyrosine kinase Bcr-Abl, which is responsible for most cases of chronic myeloid leukaemia. In addition to Bcr-Abl, bosutinib and dasatinib are potent inhibitors of many other protein tyrosine kinases including members of the Src and Tec families [20]. It follows that most of the anti-inflammatory properties of dasatinib, including the blockade of pro-inflammatory cytokine production, have been inferred to be the result of the inhibition of one or more of these protein tyrosine kinases [21–24]. Our data clearly show that the effects of these drugs on macrophage polarization are mediated by the SIKs. First, bosutinib and dasatinib potently inhibit the SIKs *in vitro* with comparable IC_50_ values to protein tyrosine kinases, in line with recent studies using chemical proteomics and *in vitro* screening approaches to characterize the targets of clinically approved anti-cancer drugs [20,25–27]. Secondly, treatment of macrophages with bosutinib and dasatinib leads to the dephosphorylation of direct substrates of the SIKs including CRTC3, which drives IL-10 production in ‘regulatory’-like macrophages. Thirdly, related drugs that are also used for the treatment of CML but do not inhibit the SIKs, such as imatinib, fail to induce CREB-dependent gene transcription and to elevate IL-10 production in macrophages. Finally, expression of a drug-resistant mutant of SIK2 in macrophages abrogates the effects of bosutinib and dasatinib on IL-10 production. Our data provide strong evidence that the mode of action of bosutinib and dasatinib in promoting the formation of ‘regulatory’-like macrophages is through the inhibition of the SIKs. Moreover, our study also highlights the important contributions that protein serine/threonine kinases make to the overall activities of protein tyrosine kinase inhibitors in cells and thus, the need to re-evaluate conclusions derived from experimental data generated using non-selective protein tyrosine kinase inhibitors. It further raises the spectre that functions in the immune system attributed to protein tyrosine kinases using dasatinib and other related compounds may, in fact, represent new functions of the SIKs.

Our data also show that the SIKs regulate macrophage polarization by fine-tuning the functions of transcription factors in these cells, rather than playing an essential role in the activation of the core signalling nodes triggered upon ligation of TLRs. Indeed, we found that the TLR-stimulated phosphorylation of ERK1/2, JNK1/2 and p38α/β MAPKs, MSK1/2, the canonical IKKs, IKKα/β, the IKK-related kinase, TBK1 and their direct substrates occurred normally in macrophages treated with different inhibitors of the SIKs including bosutinib, dasatinib and HG-9-91-01 (Figure 4 and Supplementary Figures S2 and S5). Instead, the SIKs phosphorylate and inactivate proteins that balance the functions of CREB and NF-κB. The SIKs are maintained in a constitutively active state in quiescent macrophages and their activities remain unaltered after stimulation of macrophages with TLR agonists. As a consequence, the stoichiometry of phosphorylation of their substrates, such as CRTC3, is very high in macrophages and is unaffected by TLR stimulation of macrophages [13]. Phosphorylation of CRTC3 provides a docking site for 14-3-3 proteins that sequester CRTC3 in the cytosol [19]. Treatment of macrophages with SIK inhibitors leads to the dephosphorylation of CRTC3 and its translocation to the nucleus where it can bind to CREB and enhance CREB-dependent gene transcription [13]. This SIK-CRTC3 pathway synergizes with the MSK1/2-CREB[pSer^133^] pathway to drive the polarization of macrophages towards a ‘regulatory’-like anti-inflammatory phenotype [12]. Similarly, the SIKs may also fine-tune NF-κB function in macrophages by regulating the function of histone deacetylase 4 (HDAC4), a class II HDAC. It has been proposed that inhibition of the SIKs leads to the dephosphorylation of HDAC4 in macrophages and its translocation to the nucleus where it deacetylates the NF-κB subunit p65 leading to repression of inflammatory gene expression such as TNF and IL-12 [28]. This concept helps explain the dramatic effects of SIK inhibitors on macrophage polarization.

The lack of effect of bosutinib and dasatinib on the activation of the core elements of TLR signalling pathways in bone-marrow-derived macrophages indicates that the group of protein tyrosine kinases inhibited by these drugs are not rate-limiting in these cells. Despite completely blocking Bcr-Abl, Src and Btk in primary mouse macrophages using bosutinib and dasatinib, these drugs had no effect on the activation of the MAPK cascades or the transcription factors NF-κB and IRF3 in response to LPS or Pam_3_CSK_4_. Although our findings are in agreement with other investigators who used distinct inhibitors, several studies have suggested that Src and Btk are required for membrane-proximal TLR signalling events by phosphorylating receptors and adaptor proteins [29–31]. The different outcomes in studies investigating the role of protein tyrosine kinases in innate immunity is most likely to be explained by differences in cell types and stimuli used by the investigators. It is now apparent that the roles of protein tyrosine kinases vary among different cell types. Although monocytes from X-linked agammaglobulinaemia (XLA) patients, which have lost Btk expression, fail to produce TNF in response to LPS stimulation, differentiation of these cells to macrophages using M-CSF now allows them to produce TNF at levels similar to cells from normal healthy donors [32]. Collectively, the data suggest that various protein tyrosine kinases including Src and Btk play a minimal role in M-CSF derived macrophages.

The ability of bosutinib and dasatinib to manipulate macrophage fate towards an anti-inflammatory phenotype by inhibiting the SIKs has several implications for the treatment of chronic inflammatory and autoimmune diseases in the clinic. Although the present report provides early evidence of potential anti-inflammatory actions of bosutinib, dasatinib has been shown by others to prevent the development of the cardinal features of disease in experimental models of inflammatory arthritis and allergic contact dermatitis [33,34]. Dasatinib has also been found to reduce neuro-inflammation in mouse models of Alzheimer's disease [35]. Our data propose that the anti-inflammatory properties of dasatinib in pre-clinical models may be explained, at least in part, by the inhibition of the SIKs. One potential limitation of this therapeutic strategy is that, based on studies linking the SIKs with glucose and fat metabolism [36–38], it is currently anticipated that patients treated with inhibitors of the SIKs would develop complications due to hyperglycaemia and a condition reminiscent of Type 2 diabetes. However, several patients suffering from chronic myeloid leukaemia and Type 2 diabetes show a marked improvement in their glucose handling and completely stop administering exogenous insulin shortly after initiating their dasatinib-based therapy for the treatment of imatinib-resistant leukaemia [39,40]. It is tempting to speculate that the beneficial effects of dasatinib on Type 2 diabetes is due to its anti-inflammatory properties since it has been shown that the presence of pro-inflammatory macrophages in adipose tissue is required for the development of insulin-resistance and Type 2 diabetes [41]. These observations, together with the fact that bosutinib and dasatinib are well-tolerated drugs, provides confidence that potent and selective SIK inhibitors will be well-tolerated in human patients and be efficacious in the treatment of chronic inflammatory and autoimmune diseases without overtly destabilizing glucose and fat metabolism. These new drugs could potentially also be used in the clinic for the treatment of other diseases whose pathogenesis is influenced by the inflammatory response, such as neurodegenerative, cardiovascular and metabolic diseases. In the absence of selective SIK inhibitors, bosutinib and dasatinib should be considered for the treatment of debilitating inflammatory diseases, in particular, those diseases for which there are no reasonable current treatments.

## Online data

Supplementary data
