# Supplementary material for: The clinically approved drugs dasatinib and bosutinib induce anti-inflammatory macrophages by inhibiting the salt-inducible kinases
Source: Biochem J. 2015 Jan 6;465(Pt 2):271–9. doi: 10.1042/BJ20141165 (PMC4286194; doi:10.1042/BJ20141165)
Supplement: Supplementary data [file bj4650271ntsadd.pdf]

SUPPLEMENTARY FIGURE LEGENDS

Supplementary Figure S1. Effect of Bosutinib and Dasatinib on CREB-dependent gene transcription. BMDMs were treated with vehicle control, 3 μM Bosutinib or 0.3 μM Dasatinib for 1 h and then stimulated with LPS for the indicated times. Expression of IL-10 and Nurr77 were measured by qPCR. mRNA levels were normalised to 1 in unstimulated cells (mean ± SEM, n=4).

Supplementary Figure S2. Bosutinib and Dasatinib do not affect the Pam_3CSK_4-stimulated phosphorylation of CREB. BMDMs were treated with vehicle control, 3 μM Bosutinib, 0.3 μM Dasatinib or 0.5 μM HG-9-91-01 for 1 h and then stimulated with Pam_3CSK_4 for 0, 10, 30 or 60 min. Cell lysates were immunoblotted using the indicated antibodies.

Supplementary Figure S3. Effect of Bosutinib and Dasatinib on the kinetics of cytokine secretion in mouse macrophages. Bone marrow-derived macrophages were treated with 3000 nM Bosutinib or 300 nM Dasatinib for 1 h and then stimulated for the times indicated with 100 ng/ml LPS. The concentration of **(A)** IL-10, **(B)** TNFα, **(C)** IL-6 and **(D)** IL-12p40 released into the cell culture supernatant was measured using the Bioplex system (Bio-Rad) (mean ± SEM, n=4).

Supplementary Figure S4. Induction of 'regulatory'-like macrophages by Bosutinib and Dasatinib in response to Pam_3CSK_4. (A) Effect of Bosutinib and Dasatinib on cytokine secretion. Bone marrow-derived macrophages were treated with vehicle control, 3 μM Bosutinib or 0.3 μM Dasatinib for 1 h and then stimulated with Pam_3CSK_4 for 8 h. The concentration of the different cytokines in the culture supernatant was measured using the BIOPLEX system. The data are depicted as the fold-change in cytokine secretion in the presence of the drug (n=4, mean ± SEM). Statistical significance was determined by comparing each data set to 0 using one sample t-test. **(B)** Effect of Bosutinib and Dasatinib on markers of 'regulatory'-like macrophages. BMDMs were treated with vehicle control, 3 μM Bosutinib or 0.3 μM Dasatinib for 1h and then stimulated with Pam_3CSK_4 for 4 h (SPHK1, LIGHT) or 8 h (Arg1) prior to mRNA extraction. Gene expression was measured by qPCR. mRNA levels were normalised to 1 in unstimulated cells (mean ± SEM, n=4).

Supplementary Figure S5. Bosutinib and Dasatinib do not affect the TLR-stimulated activation of NFκB and IRF3 pathways. BMDMs were treated with vehicle control, 3 μM Bosutinib, 0.3 μM Dasatinib or 0.5 μM HG-9-91-01 for 1 h and then stimulated with **(A,B,C)** LPS or **(D,E)** Pam_3CSK_4 for 0, 10, 30 or 60 min. Cell lysates were immunoblotted using the indicated antibodies. The panels depict the following signaling cascades: A and D- activation of NFκB; B-activation of IRF3; C and E, activity of selected protein tyrosine kinases.

Supplementary Table S1- Kinase profiling of Bosutinib and Dasatinib

	Bosutinib		Dasatinib	
Kinase	0.1 µM	1 µM	0.1 µM	1 µM
ABL	1 ± 0	0 ± 0	0 ± 0	0 ± 0
AMPK	65 ± 4	26 ± 2	107 ± 13	120 ± 5
ASK1	96 ± 9	106 ± 1	108 ± 7	118 ± 7
Aurora A	95 ± 2	98 ± 6	94 ± 0	101 ± 1
Aurora B	93 ± 13	50 ± 7	120 ± 10	108 ± 10
BRK	88 ± 11	48 ± 0	5 ± 0	2 ± 0
BRSK1	101 ± 1	112 ± 5	126 ± 0	123 ± 1
BRSK2	107 ± 12	120 ± 4	102 ± 14	108 ± 13
BTK	1 ± 0	1 ± 0	1 ± 0	1 ± 0
CAMK1	78 ± 11	74 ± 16	89 ± 16	77 ± 1
CAMKK beta	81 ± 2	27 ± 0	96 ± 4	99 ± 19
CDK2-Cyclin A	110 ± 32	103 ± 5	116 ± 1	112 ± 1
CDK9-Cyclin T1	101 ± 8	125 ± 16	122 ± 8	138 ± 12
CHK1	91 ± 2	57 ± 0	100 ± 6	96 ± 6
CHK2	37 ± 0	10 ± 2	98 ± 8	102 ± 1
CK1 delta	86 ± 20	60 ± 7	113 ± 4	124 ± 1
CK1 gamma 2	117 ± 1	108 ± 4	115 ± 0	113 ± 12
CK2	77 ± 3	106 ± 1	95 ± 3	80 ± 28
CLK2	75 ± 9	23 ± 0	120 ± 6	121 ± 6
CSK	65 ± 7	4 ± 0	97 ± 19	8 ± 3
DAPK1	92 ± 3	105 ± 4	126 ± 10	102 ± 7
DDR2	22 ± 1	2 ± 0	1 ± 1	0 ± 0
DYRK1A	91 ± 3	96 ± 13	111 ± 4	126 ± 19
DYRK2	71 ± 4	87 ± 5	101 ± 5	117 ± 9
DYRK3	85 ± 11	90 ± 16	98 ± 7	104 ± 9
EF2K	81 ± 2	95 ± 6	104 ± 4	95 ± 5
EIF2AK3	107 ± 5	103 ± 0	106 ± 6	113 ± 2
EPH-A2	4 ± 0	1 ± 0	1 ± 0	1 ± 0
EPH-A4	3 ± 1	1 ± 0	2 ± 0	2 ± 2
EPH-B1	12 ± 0	0 ± 2	-1 ± 1	-1 ± 2
EPH-B2	1 ± 1	0 ± 0	1 ± 0	1 ± 0
EPH-B3	16 ± 0	1 ± 0	1 ± 0	1 ± 0
EPH-B4	10 ± 0	2 ± 0	20 ± 0	3 ± 0
ERK1	98 ± 5	103 ± 1	91 ± 6	114 ± 12
ERK2	104 ± 18	103 ± 15	93 ± 9	102 ± 1
ERK5	74 ± 6	67 ± 10	87 ± 2	91 ± 4
ERK8	96 ± 13	87 ± 3	90 ± 14	95 ± 11
FGF-R1	94 ± 4	74 ± 6	88 ± 7	84 ± 6
GCK	9 ± 2	4 ± 0	95 ± 7	56 ± 8
GSK3 beta	109 ± 6	114 ± 22	103 ± 2	121 ± 12
HER4	9 ± 0	1 ± 1	27 ± 1	6 ± 0
HIPK1	95 ± 0	57 ± 0	105 ± 4	122 ± 6
HIPK2	71 ± 7	42 ± 4	95 ± 1	97 ± 5
HIPK3	102 ± 14	66 ± 5	102 ± 3	113 ± 7
IGF-1R	89 ± 8	79 ± 4	107 ± 6	98 ± 7
IKK beta	92 ± 15	86 ± 8	97 ± 21	99 ± 9
IKK epsilon	46 ± 7	6 ± 0	102 ± 5	123 ± 13
IR	87 ± 7	107 ± 34	99 ± 2	94 ± 7
IRAK1	77 ± 3	65 ± 0	96 ± 1	108 ± 3
IRAK4	55 ± 3	23 ± 0	112 ± 24	102 ± 6
IRR	72 ± 5	59 ± 1	89 ± 4	90 ± 14
JAK2	72 ± 5	26 ± 0	94 ± 2	42 ± 1
JNK1	88 ± 15	102 ± 1	108 ± 4	121 ± 9
JNK2	108 ± 20	108 ± 11	110 ± 10	125 ± 0
JNK3	102 ± 3	106 ± 10	108 ± 0	113 ± 15
Lck	1 ± 0	1 ± 0	1 ± 0	1 ± 0
LKB1	84 ± 13	104 ± 15	96 ± 18	100 ± 14
MAP4K3	3 ± 0	1 ± 0	88 ± 15	54 ± 1
MAP4K5	0 ± 2	1 ± 0	22 ± 1	3 ± 1
MAPKAP-K2	87 ± 5	94 ± 5	114 ± 4	111 ± 7
MAPKAP-K3	113 ± 1	103 ± 8	117 ± 3	132 ± 1
MARK1	84 ± 5	76 ± 4	115 ± 3	127 ± 3
MARK2	89 ± 8	68 ± 1	118 ± 4	126 ± 2
MARK3	74 ± 10	41 ± 3	96 ± 16	104 ± 5
MARK4	101 ± 7	72 ± 5	105 ± 12	115 ± 24
MEKK1	95 ± 5	85 ± 6	98 ± 4	102 ± 1
MELK	95 ± 10	66 ± 1	112 ± 7	128 ± 7
MINK1	5 ± 1	1 ± 0	90 ± 4	51 ± 1
MKK1	81 ± 2	31 ± 9	118 ± 10	134 ± 16
MKK2	60 ± 3	21 ± 4	113 ± 11	91 ± 0

	Bosutinib		Dasatinib	
Kinase	0.1 µM	1 µM	0.1 µM	1 µM
MKK6	92 ± 6	115 ± 5	104 ± 11	112 ± 4
MLK1	52 ± 9	18 ± 4	72 ± 1	41 ± 2
MLK3	42 ± 4	8 ± 1	76 ± 10	39 ± 6
MNK1	99 ± 14	105 ± 2	108 ± 0	102 ± 9
MNK2	87 ± 6	100 ± 9	97 ± 8	106 ± 2
MPSK1	117 ± 15	110 ± 11	101 ± 5	123 ± 1
MSK1	93 ± 2	102 ± 12	94 ± 4	103 ± 1
MST2	65 ± 6	17 ± 2	75 ± 2	81 ± 6
MST3	68 ± 0	21 ± 4	114 ± 20	93 ± 2
MST4	20 ± 1	7 ± 0	97 ± 10	77 ± 4
NEK2a	76 ± 4	63 ± 2	122 ± 11	98 ± 4
NEK6	102 ± 1	111 ± 3	113 ± 4	123 ± 6
NUAK1	63 ± 12	21 ± 2	119 ± 10	118 ± 8
OSR1	96 ± 3	65 ± 2	100 ± 1	101 ± 0
p38 alpha MAPK	97 ± 4	72 ± 3	79 ± 15	29 ± 2
p38 beta MAPK	94 ± 15	108 ± 24	105 ± 11	44 ± 4
p38 delta MAPK	111 ± 12	116 ± 12	118 ± 17	133 ± 1
p38 gamma MAPK	88 ± 3	109 ± 5	100 ± 4	105 ± 7
PAK2	115 ± 6	108 ± 1	118 ± 2	119 ± 12
PAK4	110 ± 16	126 ± 1	137 ± 21	132 ± 16
PAK5	105 ± 18	129 ± 5	131 ± 12	138 ± 7
PAK6	103 ± 11	132 ± 7	126 ± 9	120 ± 4
PDGFRA	96 ± 1	79 ± 0	6 ± 1	3 ± 1
PDK1	92 ± 16	98 ± 0	94 ± 9	92 ± 1
PHK	55 ± 0	12 ± 0	101 ± 10	103 ± 1
PIM1	91 ± 7	94 ± 0	90 ± 1	98 ± 1
PIM2	99 ± 0	96 ± 8	104 ± 3	105 ± 1
PIM3	92 ± 16	101 ± 3	96 ± 8	108 ± 5
PINK	112 ± 9	125 ± 13	117 ± 1	133 ± 5
PKA	111 ± 16	105 ± 7	110 ± 12	121 ± 27
PKB alpha	110 ± 16	116 ± 30	103 ± 15	120 ± 13
PKB beta	102 ± 6	90 ± 3	94 ± 11	94 ± 2
PKC alpha	96 ± 5	103 ± 3	98 ± 3	105 ± 14
PKC gamma	88 ± 6	102 ± 5	107 ± 22	114 ± 22
PKC zeta	91 ± 2	102 ± 4	101 ± 3	109 ± 7
PKD1	89 ± 2	86 ± 8	92 ± 21	107 ± 15
PLK1	97 ± 12	74 ± 6	133 ± 18	99 ± 15
PRAK	97 ± 2	125 ± 1	129 ± 14	128 ± 7
PRK2	86 ± 6	56 ± 3	100 ± 2	101 ± 0
RIPK2	86 ± 2	45 ± 1	5 ± 0	10 ± 12
ROCK 2	99 ± 6	74 ± 7	130 ± 10	132 ± 7
RSK1	90 ± 8	70 ± 3	105 ± 2	102 ± 5
RSK2	86 ± 6	64 ± 1	86 ± 9	101 ± 3
S6K1	98 ± 1	86 ± 2	108 ± 3	113 ± 11
SGK1	98 ± 20	100 ± 2	87 ± 11	96 ± 7
SIK2	8 ± 0	3 ± 0	4 ± 1	2 ± 1
SIK3	9 ± 0	2 ± 0	9 ± 2	2 ± 0
SmMLCK	88 ± 2	27 ± 1	111 ± 4	111 ± 7
Src	1 ± 0	1 ± 0	1 ± 0	2 ± 0
SRPK1	89 ± 6	109 ± 5	104 ± 9	107 ± 9
STK33	34 ± 0	17 ± 4	105 ± 6	110 ± 4
SYK	77 ± 2	39 ± 1	97 ± 1	90 ± 0
TAK1	41 ± 9	5 ± 0	97 ± 23	99 ± 7
TAO1	85 ± 8	102 ± 7	120 ± 9	95 ± 7
TBK1	75 ± 1	29 ± 1	100 ± 1	97 ± 10
TESK1	82 ± 15	61 ± 2	50 ± 3	8 ± 0
TGFBR1	87 ± 14	101 ± 6	103 ± 1	82 ± 5
TIE2	74 ± 3	64 ± 3	88 ± 7	78 ± 5
TLK1	63 ± 13	35 ± 3	96 ± 4	124 ± 0
TrkA	25 ± 2	7 ± 0	107 ± 11	88 ± 2
TSSK1	67 ± 1	22 ± 2	106 ± 11	113 ± 3
TTBK1	95 ± 2	114 ± 3	112 ± 8	112 ± 1
TTBK2	87 ± 6	92 ± 6	110 ± 5	117 ± 7
TTK	94 ± 0	99 ± 7	104 ± 6	109 ± 9
ULK1	79 ± 5	44 ± 2	95 ± 9	101 ± 0
ULK2	78 ± 10	37 ± 1	101 ± 3	91 ± 3
VEGFR1	88 ± 5	54 ± 3	78 ± 9	53 ± 4
WNK1	102 ± 2	104 ± 9	104 ± 4	104 ± 12
YES1	2 ± 1	1 ± 0	1 ± 0	1 ± 0
ZAP70	99 ± 4	109 ± 18	109 ± 1	121 ± 27

RESULTS SHOW MEAN % ACTIVITY REMAINING AND STANDARD DEVIATION

Supplementary Figure S1

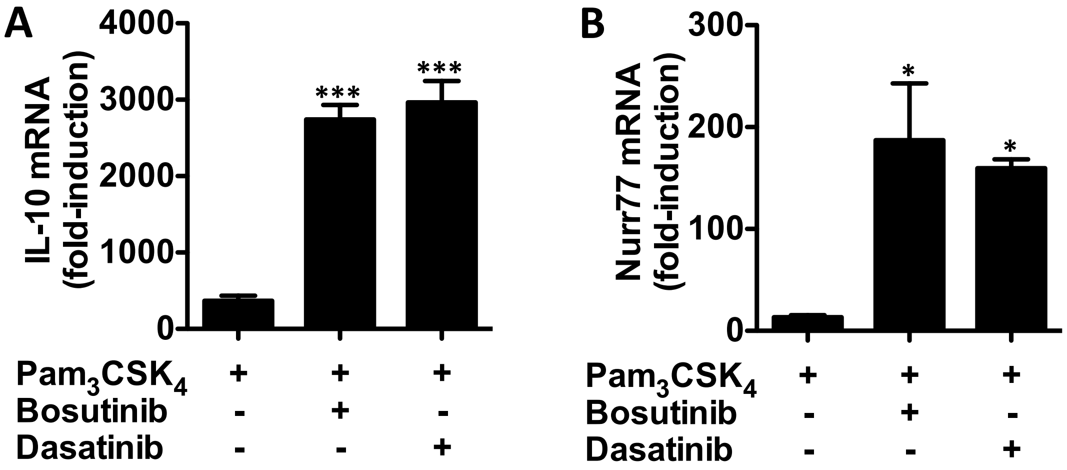

Ozanne *et al.*

Supplementary Figure S2

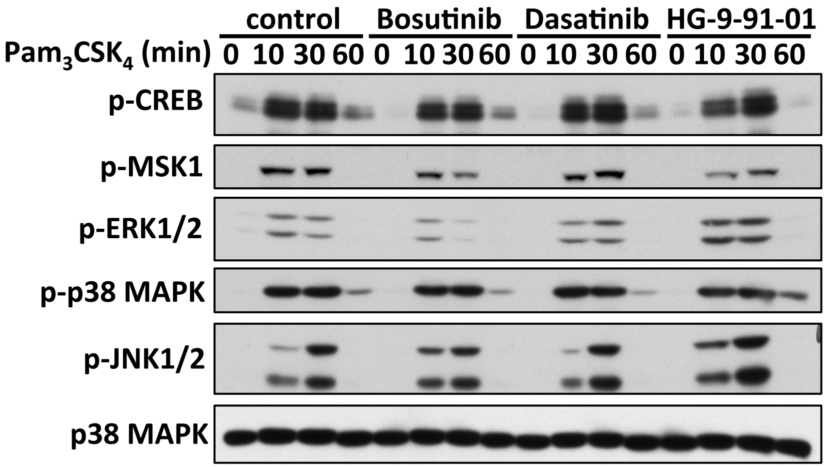

Ozanne *et al.*

Supplementary Figure S3

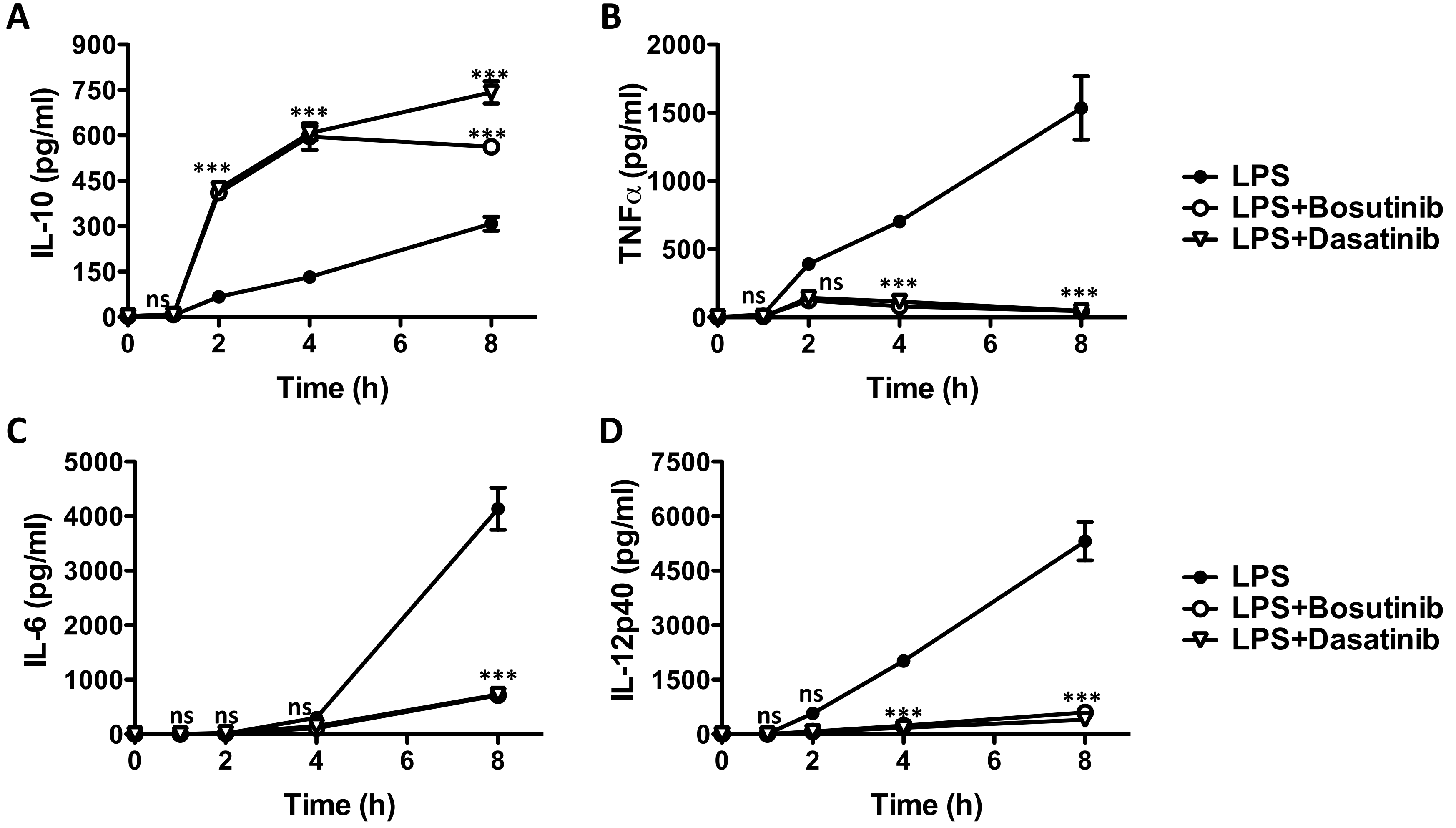

Ozanne *et al.*

Supplementary Figure S4

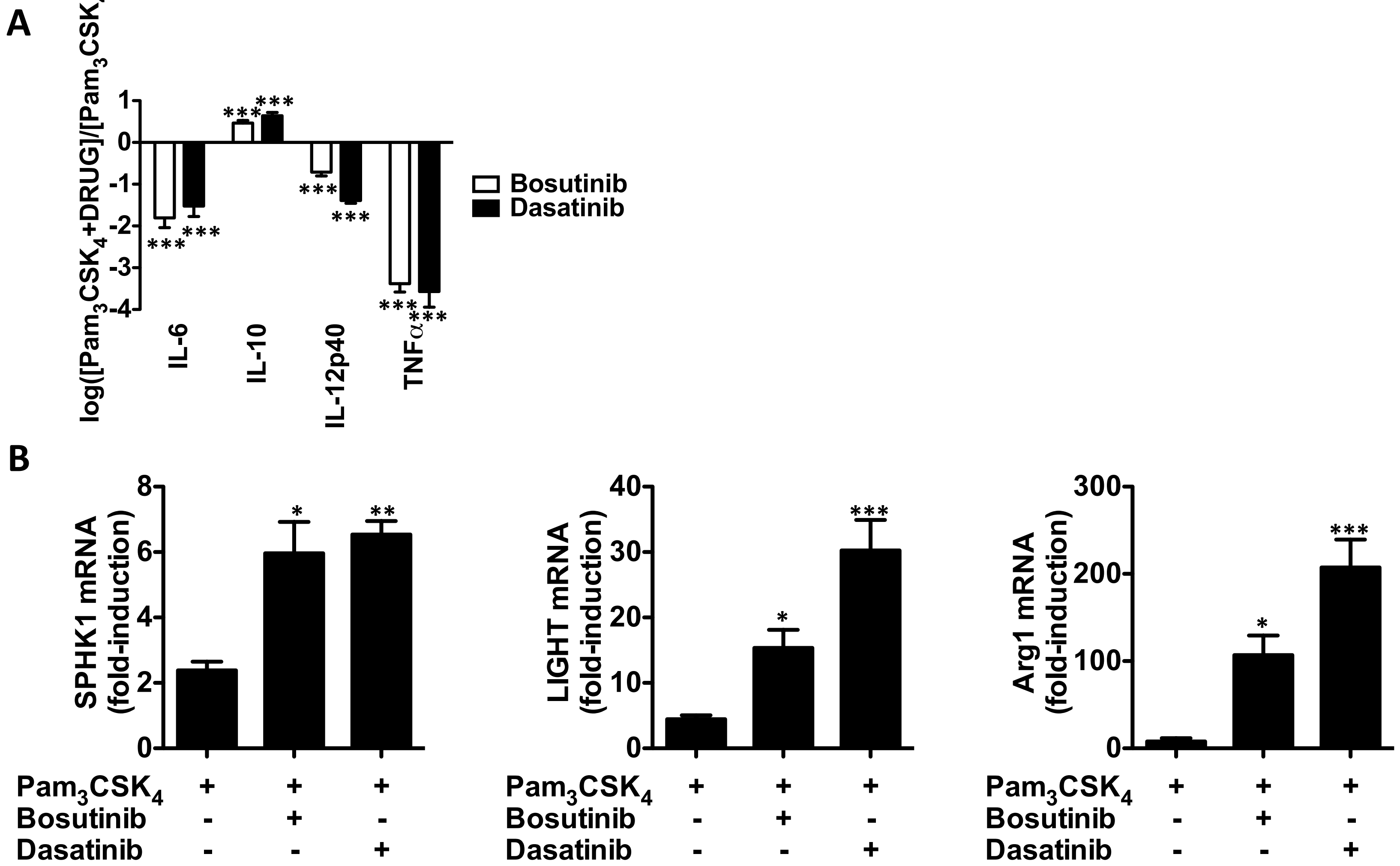

Ozanne *et al.*

Supplementary Figure S5

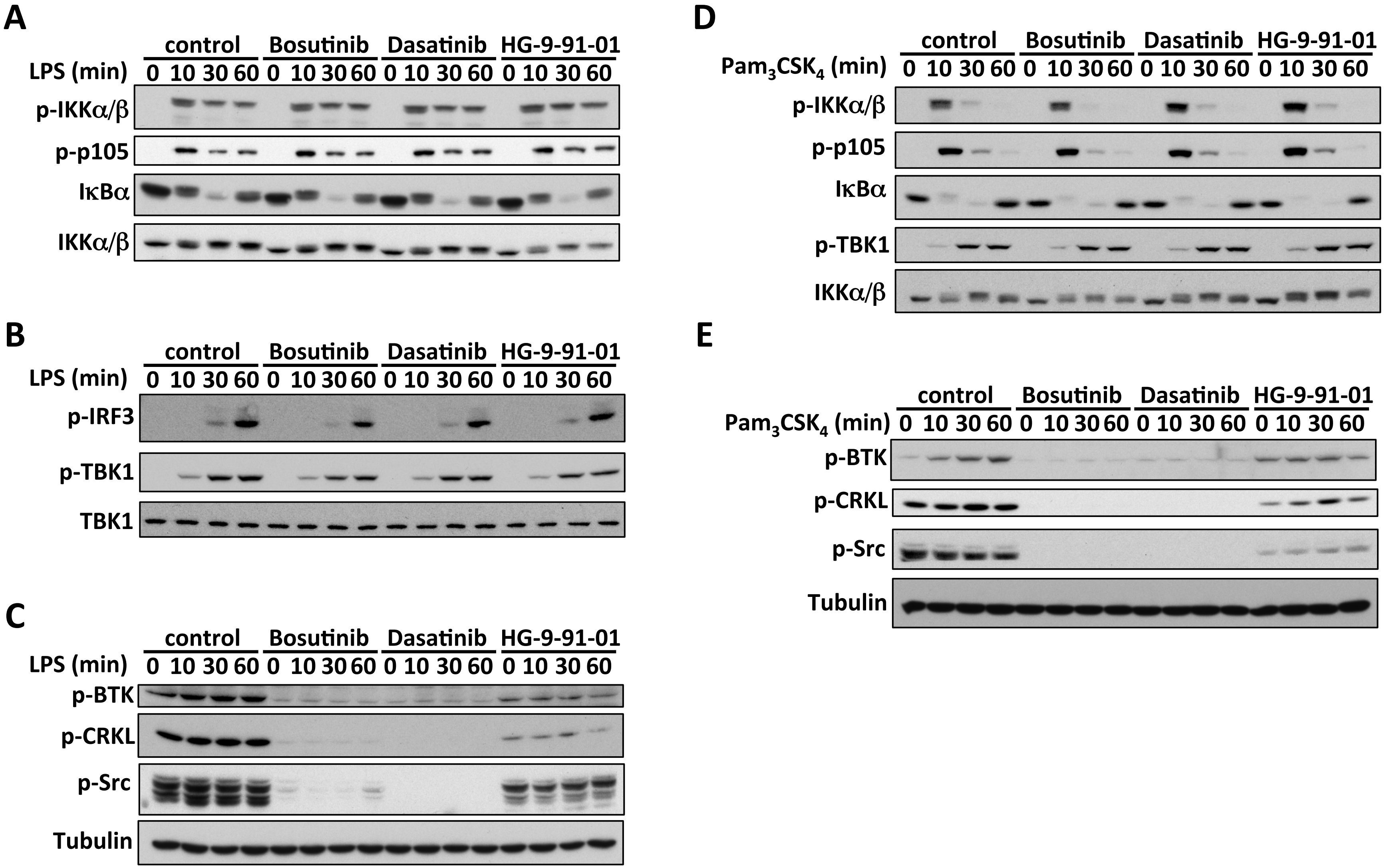

Ozanne *et al.*
